# BMP4 and Temozolomide Synergize in the Majority of Patient-Derived Glioblastoma Cultures

**DOI:** 10.3390/ijms251810176

**Published:** 2024-09-22

**Authors:** Iris S. C. Verploegh, Andrea Conidi, Hoesna El Hassnaoui, Floor A. M. Verhoeven, Anne L. Korporaal, Ioannis Ntafoulis, Mirjam C. G. N. van den Hout, Rutger W. W. Brouwer, Martine L. M. Lamfers, Wilfred F. J. van IJcken, Danny Huylebroeck, Sieger Leenstra

**Affiliations:** 1 Department of Neurosurgery, Erasmus University Medical Center, 3015 GD Rotterdam, The Netherlands; i.verploegh@erasmusmc.nl (I.S.C.V.);; 2 Department of Cell Biology, Erasmus University Medical Center, 3015 GD Rotterdam, The Netherlands; 3 Department of Clinical Genetics, Erasmus University Medical Center, 3015 GD Rotterdam, The Netherlands; 4 Center for Biomics, Erasmus University Medical Center, 3015 GD Rotterdam, The Netherlands

**Keywords:** bone morphogenetic proteins, drug synergy, glioblastoma, temozolomide, therapy

## Abstract

One of the main causes of poor prognoses in patient with glioblastoma (GBM) is drug resistance to current standard treatment, which includes chemoradiation and adjuvant temozolomide (TMZ). In addition, the concept of cancer stem cells provides new insights into therapy resistance and management also in GBM and glioblastoma stem cell-like cells (GSCs), which might contribute to therapy resistance. Bone morphogenetic protein-4 (BMP4) stimulates astroglial differentiation of GSCs and thereby reduces their self-renewal capacity. Exposure of GSCs to BMP4 may also sensitize these cells to TMZ. A recent phase I trial has shown that local delivery of BMP4 is safe, but a large variation in survival is seen in these treated patients and in features of their cultured tumors. We wanted to combine TMZ and BMP4 (TMZ + BMP4) therapy and assess the inter-tumoral variability in response to TMZ + BMP4 in patient-derived GBM cultures. A phase II trial could then benefit a larger group of patients than those treated with BMP4 only. We first show that simultaneous treatment with TMZ + BMP4 is more effective than sequential treatment. Second, when applying our optimized treatment protocol, 70% of a total of 20 GBM cultures displayed TMZ + BMP4 synergy. This combination induces cellular apoptosis and does not inhibit cell proliferation. Comparative bulk RNA-sequencing indicates that treatment with TMZ + BMP4 eventually results in decreased MAPK signaling, in line with previous evidence that increased MAPK signaling is associated with resistance to TMZ. Based on these results, we advocate further clinical trial research to test patient benefit and validate pathophysiological hypothesis.

## 1. Introduction

Glioblastoma (GBM) is the most common and lethal primary tumor of the central nervous system [1]. Current standard therapy involving surgical resection followed by chemotherapy with temozolomide (TMZ) and radiotherapy does not lead to tumor remission. Because of the high invasive growth of the tumor into the brain parenchyma surrounding the tumor, complete resection is not possible. In addition, the intact blood–brain barrier in peritumoral areas causes delivery problems for many compounds while the high cellular heterogeneity, which is a hallmark of GBM and its glioblastoma stem cell-like cells (GSCs), results in selection and subsequent expansion of resistant tumor cell clusters after chemotherapy. As a result, patients with GBM have a dismal prognosis, with a median survival of approximately 18 months only [2].

GSCs are among the culprits for cellular heterogeneity and chemotherapy resistance [3]. Analogous to embryonic, early-post natal and, e.g., adult forebrain ventricular zone neural stem cells (NSCs), GSCs can self-renew and differentiate into several neural lineages [4]. Induced astroglial differentiation of GSCs in particular is considered a potential mechanism for counteracting the aggressiveness of this subpopulation of GBM tumor cells. Bone morphogenetic protein-4 (BMP4) is a growth-differentiation factor of the transforming growth factor type-β (TGFβ) family of ligands. In the later phases of central nervous system development in embryos and in adult neurogenesis, BMP4 co-controls and stimulates differentiation of the residing NSCs along the astroglial lineage [5]. These findings lead to the hypothesis of BMPs being an interesting agent for targeting GSCs in GBM. Studies in glioma cell cultures and in vivo have already shown that BMP4 indeed induces the differentiation of GSCs together with decreased cell proliferation and apoptosis within the tumor and increased survival in vivo [6,7]. However, studies have also reported that monotherapy with BMP4 had only temporary effects and can also induce senescence of proliferation-competent tumor cells, thus contributing to therapy resistance [8,9].

Using single-cell transcriptomics of BMP4-treated primary cell cultures of GBM tumors, we recently showed inter- and intra-tumoral heterogeneity in response to BMP4. In particular, in cultures with a large proportion of cells that show, prior to BMP4 addition, increased OLIG1/2 expression, there is decreased cell viability in response to BMP4 [4]. This implied not only that a subset of GBM patients would benefit from monotherapy with BMP4, but also that still a larger subset of such treated GBM patients would not have a survival benefit. This was reinforced by a recent phase I trial that demonstrated the safety of local delivery of BMP4 but also highlighted the variability in survival upon this treatment [10]. To optimize a future clinical trial, we wanted to assess the effectivity of combination therapy with TMZ + BMP4 in cultured tumor cells of a group of GBM patients. One hypothesis in this context is that BMP4 may sensitize otherwise therapy-resistant GSCs to TMZ. First, we optimized the combinatorial treatment protocol then we tested the most effective protocol in GBM patient-derived cell cultures and started to investigate the underlying mechanisms of TMZ + BMP4 co-action.

## 2. Results

### 2.1. Simultaneous Administration of TMZ and BMP4 to Patient-Derived GBM Cell Cultures Is More Effective in Decreasing Cell Viability than Sequential Treatment

Our hypothesis was that BMP4 may sensitize otherwise therapy-resistant GBM cells to TMZ, for BMP differentiates GSCs, which have been proposed as being responsible for therapy resistance. In particular, we first wondered whether sequential treatment with either BMP4 (to induce astroglial differentiation) and TMZ or simultaneous treatment with TMZ + BMP4 would be a more effective paradigm with significant effects on cell death and survival.

**Figure 1:**
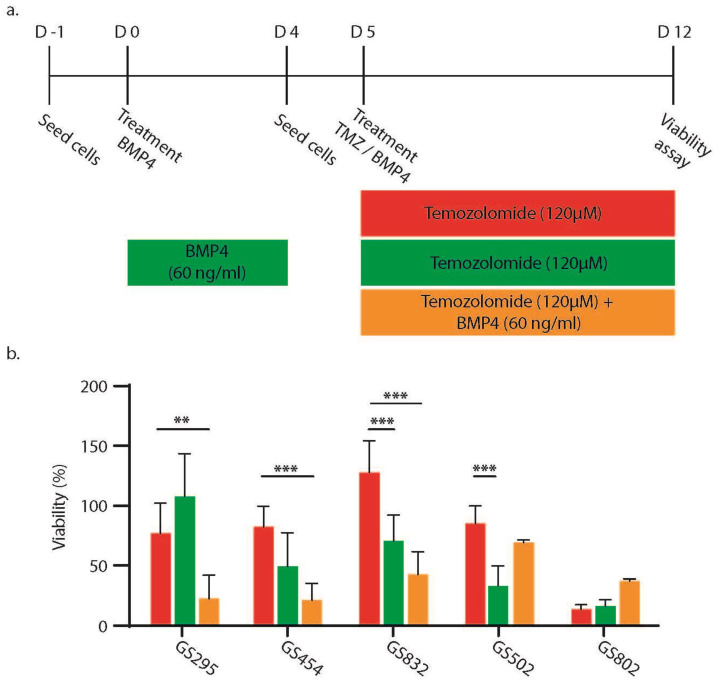
Combination therapy TMZ + BMP4 is generally more effective than sequential therapy: (**a**) schematic representation of the treatment protocol per day, starting on day-1 (D-1); (**b**) mean cell viability with standard deviation (n = 3) on day 7 of treatment normalized to untreated cells. ** *p* < 0.01, *** *p* < 0.001.

For this, we treated five patient-derived GBM cultures (see Section 4 with varying sensitivities to TMZ and BMP4 [4] either for four days with 60 ng BMP4/mL and then for seven days with 120 µM TMZ or exclusively for seven days with 120 µM TMZ or a combination (120 µM TMZ+ 60 ng BMP4/mL) of both agents. For a schematic overview of these three modes of treatment, see Figure 1a. After each of these treatments, cell viability was first assessed. Culture GS802 was highly sensitive to TMZ; therefore, this culture did not benefit from additional treatment with BMP4 (Figure 1b). In the remaining four cultures that were resistant to treatment with TMZ (defined as having an EC50 > 100 µM for this agent [11]), the addition of BMP4 significantly reduced cell viability. This reduction was generally the largest after the simultaneous administration of TMZ + BMP4, with the exception of cultured tumor GS502 (Figure 1b).

### 2.2. BMP4 Synergizes with TMZ in the Majority of Patient-Derived GBM Cultures

We also observed inter-tumoral heterogeneity in cell viability in response to TMZ + BMP4 treatment, as seen previously with BMP4 and TMZ monotherapy (see Figure 1b) [4,12]. Therefore, we wondered what proportion of tumors would benefit from this combinatorial treatment and whether a different dose of either component might be more effective.

**Figure 2:**
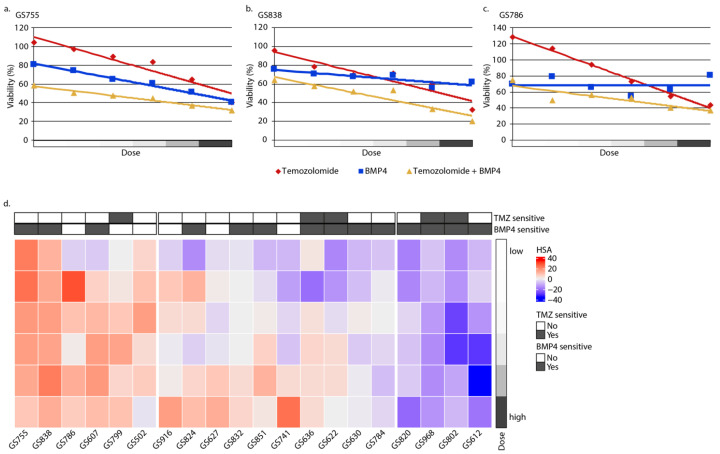
GBM cell culture viability after treatment with temozolomide (TMZ) and BMP4. Average cell viability (n = 3) in cultures GS755 (**a**), GS838 (**b**), and GS786 (**c**) after 7 days of treatment with a 3-fold dilution ranging from 180 ng down to 0.74 ng BMP4/mL (blue) and 360 µM down to 1.5 µM TMZ (red) and a combination of both (yellow). The lowest dose is depicted in white (1.5 µM TMZ; 0.75 ng BMP4/mL) increasing (4.5 µM/2.25 ng/mL; 13.5 µM/6.8 ng/mL; 40 µM/20 ng/mL; 120 µM/60 ng/mL) to the highest dose depicted in dark grey (360 µM TMZ and 180 ng BMP4/mL). (**d**) Heat map of HSA score per dose and culture. A low HSA score (antagonism) is depicted in blue and a high score (synergy) is shown in red. The bars above the figure represent the sensitivity (black) or resistance (white) to TMZ (EC50 > 100 µM) and BMP4 (EC50 > 60 ng/mL).

For this, we expanded our analyses to a panel of 20 patient-derived GBM cultures (see Supplementary Table S1) and treated these with TMZ, BMP4, and the TMZ + BMP4 simultaneous combination. We achieved this applying three-fold dilutions (from 180 ng down to 0.74 ng BMP4/mL; and from 360 µM down to 1.5 µM of TMZ). The highest doses were not directly associated with increased synergy in the cell viability assay, but doses of 60 ng BMP/mL [4] and 120 µM of TMZ, as used in the cell viability assay, accomplished synergy in most of the cultures (Figure 2a–c), e.g., in GS755, GS838, and GS786.

The amplitude of this synergy was, however, highly variable among the different cultures: in approximately one-third of the tested cultures (i.e., GS755 to GS502) TMZ + BMP4 synergized at almost any dose, whereas in 40% of the cultures (GS916 to GS622), this synergy was only present after treatment with the highest respective doses. In addition, in 10% of the cultures the combination merely had an additive effect (GS630 and GS784), whereas in the remaining four cultures (GS820 to GS612) TMZ + BMP4 even showed an antagonistic effect. Furthermore, the amplitude of synergy was also not related to sensitivity to monotherapy with either TMZ or BMP4 (Figure 2d). As a first safety assessment, we also determined the effect of BMP4, TMZ, and TMZ + BMP4 on normal human astrocytes. At high concentrations, TMZ is cytotoxic to these cells; BMP4 does not significantly enhance this effect (Supplementary Figure S1).

Thus, in a panel of 20 patient-derived cultures, 70% (GS755 to GS622) of these showed decreased cell viability after combination treatment with 120 µM TMZ and 60 ng BMP4/mL.

### 2.3. Decreased Cell Viability after Synergistic TMZ + BMP4 Results from Increased Apoptosis

**Figure 3:**
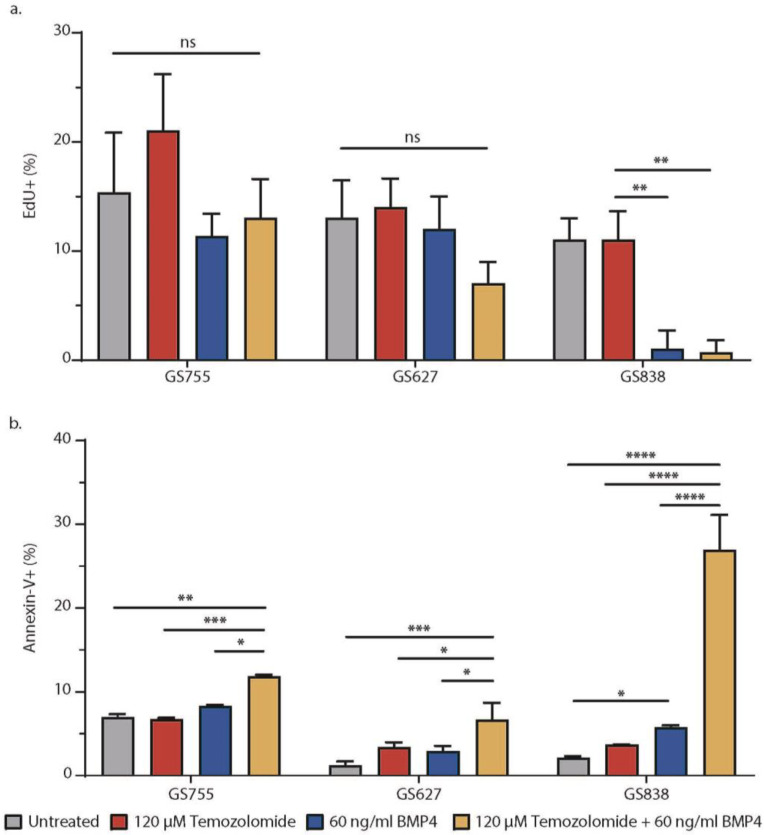
Effects of temozolomide (TMZ) and BMP4 on GBM culture cell proliferation and apoptosis. Average percentage of proliferating cells (EdU+) (**a**) and apoptotic cells (annexin-V+) (**b**) with standard deviation (n = 3) in untreated condition (gray) and after treatment with TMZ (red), BMP4 (blue), and TMZ + BMP4 (yellow). * *p* < 0.05, ** *p* < 0.01, *** *p* < 0.001, **** *p* < 0.0001, ns = not significant. No further specified comparisons were considered statistically significant.

Previous studies have shown that BMP4 by itself reduces cell proliferation in GBM tumors, whereas TMZ by itself induces apoptosis [4,12]. To document this for TMZ + BMP4, we used the EdU assay as a readout for cell proliferation and annexin-V staining for apoptotic cell numbers in three selected cultures (GS755, GS627, GS838; Figure 3). Our EdU assay results show that two days of treatment with 60 ng BMP4/mL significantly reduced cell proliferation in one of the three tested cultures; as expected, the combination with 120 µM TMZ did not significantly enhance this effect (Figure 3a). In all three tested cultures, TMZ + BMP4 significantly increased the percentage of annexin-V+ cells compared to monotherapy with either agent (Figure 3b). Therefore, the synergistic effect of TMZ and BMP4 on cell viability largely resulted from increased apoptosis.

### 2.4. Gene Expression Profiles Associated with BMP4 Monotherapy, and Not with Combination Therapy, Are Unique per Culture

**Figure 4:**
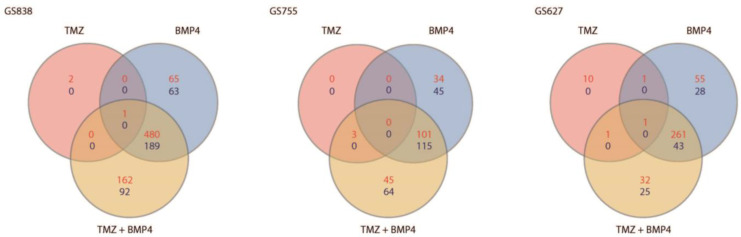
Venn diagram of DEGs (*p* < 0.01, log FC > 1.5) treated compared with untreated cells per culture, where upregulated genes are red and downregulated cells are blue.

To further investigate possible mechanisms underlying the synergy between TMZ and BMP4, we performed bulk RNA-sequencing (RNA-seq). For this, we again treated three cultures for up to 48 h in order to observe relatively long-term treatment effects in mostly viable cells and we again used TMZ, BMP4, and TMZ + BMP4. We also submitted three biological replicates for each cultured GBM tumor, which had been analyzed earlier (i.e., GS838, GS755, GS627, given in this order in Figure 4), to RNA-seq together with the respective untreated control. We determined the number of DEGs with at least a two-fold-change compared to the untreated control of each culture and a p-adjusted value of <0.01. Using these criteria for DEGs, we found that treatment with TMZ had little effect on the transcriptome of each of the GBM cultures, since it resulted in differential expression of only 3 genes in GS838, 3 in GS755, and 13 in GS627 (Figure 4).

**Figure 5:**
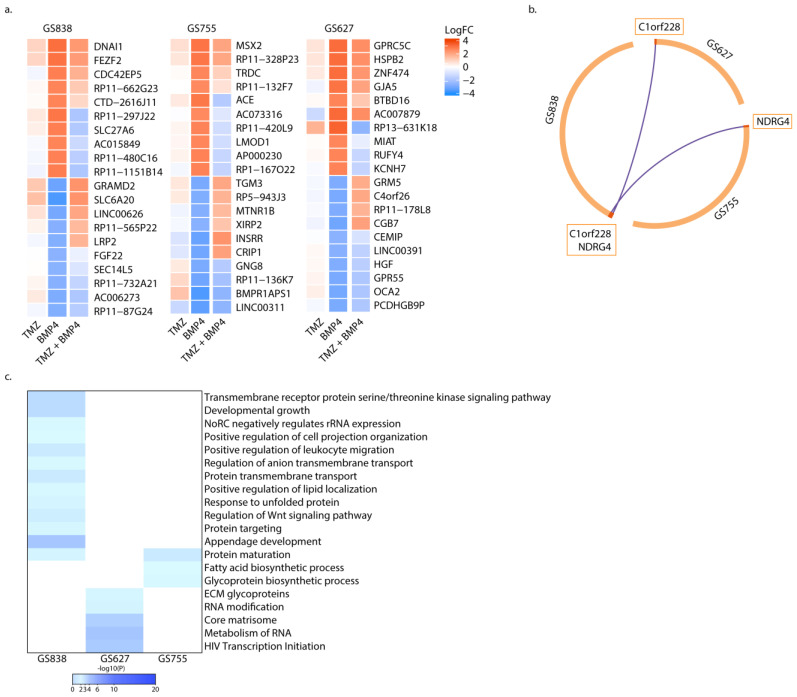
Exclusive differentially expressed genes (DEGs) after monotherapy with BMP4. (**a**) Heatmap of the top 10 most up- (red) and downregulated (blue) genes after treatment with TMZ, BMP4, and TMZ + BMP4, which are unique for monotherapy with BMP4, per sequenced culture. (**b**) Overlap of genes that were uniquely differentially expressed after monotherapy with BMP4 per culture. Purple lines linking identical genes. (**c**) Heat map of gene set enrichment analysis of DEGs unique after BMP4 monotherapy.

Interestingly, in each of these cultures, there was a set of genes that was differentially expressed compared to untreated cells after BMP4 monotherapy. Furthermore, these sets were not differentially expressed in the counterpart TMZ + BMP4 treated cultures. Indeed, in GS838, GS755, and GS627 a total of 128, 79, and 83 genes, respectively, were uniquely differentially expressed after monotherapy with BMP4 (Figure 4 and Figure 5a; Supplementary Figure S2a). Interestingly, several genes encoding ribosomal proteins (RPs) were differentially expressed in all cultures. Differential expression of RPs could be associated with cell death [13], which we would expect in these cultures that showed enhanced apoptosis after TMZ + BMP4. None of these genes were unique to BMP4 monotherapy in either culture (Figure 5b). Notably, c1orf228 (also called ARMH1) was unique in the case of monotherapy with BMP4 in GS627 and GS838, and NDRG4 was in GS755 and GS838. To the best of our knowledge, no detailed functional characterization has been performed for ARMH1, while knockdown of NDRG4 causes cell cycle arrest followed by apoptosis [14].

Since we did not identify a unique gene signature exclusively associated with BMP4 monotherapy, we performed Metascape pathway enrichment analysis (metascape.org). Only one general pathway was enriched in two out of the three tested cultures, i.e., protein maturation (based on SEC11A, APH1B, and CALR expression) (Figure 5c). Thus, in our cultures, the transcriptomic profiles and their changes that were observed after treatment with BMP4, but neither after treatment with BMP4 + TMZ nor TMZ alone, were generally unique to each culture.

### 2.5. Synergy of TMZ + BMP4 Is Associated with Upregulated BHLHE40, and Decreased MAPK Signaling and Purine Metabolism

**Figure 6:**
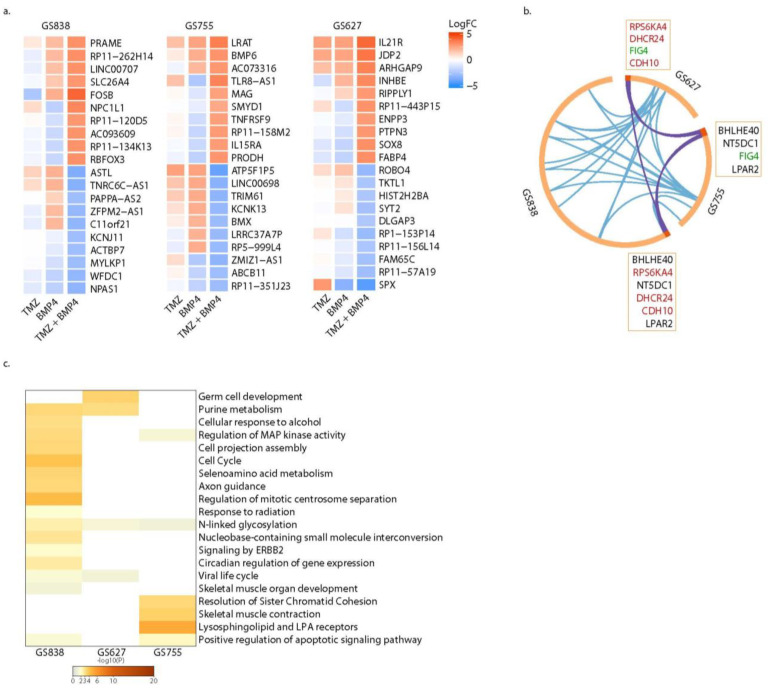
Exclusive differentially expressed genes (DEGs) after combination therapy with TMZ and BMP4. (**a**) Heat map of the top 10 most upregulated (red) and downregulated (blue) genes after treatment with TMZ, BMP4, and TMZ + BMP4, which are unique for combination therapy of TMZ + BMP4, per sequenced culture. (**b**) Overlap of genes that were uniquely differentially expressed after monotherapy with BMP4 per culture. Purple lines link identical genes and blue lines link genes that belong to similar enriched ontology terms. Genes marked in red are common between GS627 and GS838, green between GS627 and GS755, and black between GS755 and GS838. (**c**) Heatmap of gene set enrichment analysis of unique DEGs after treatment with BMP4 and TMZ.

For the set of genes that were exclusive DEGs after monotherapy with BMP4 in each culture, there was also a set of exclusive DEGs after combination treatment with TMZ + BMP4 (Figure 6a, for a display of the respective top 10 DEGs, either up- or downregulated per culture).

We hypothesized that these genes might be responsible for the synergistic interaction of both agents. The numbers of DEGs were 254, 109, and 57 for GS838, GS755, and GS627, respectively (Figure 4; Supplementary Figure S2b). None of these DEGs were shared among the three cultures. However, seven of these DEGs were present in two of the three cultures. GS838 and GS627 had only one common gene, i.e., FIG4, whose role in GBM has not yet been elucidated (Figure 6b). GS627 and GS755 shared the DEG RPS6KA4 (upregulated), DHCR24 (downregulated), and CDH10 (downregulated in GS627 and upregulated in GS755).

RPS6KA4 phosphorylates CREB1, which enhances the ability of the BMP pathway to upregulate transcription of the inhibitory-SMAD encoding gene SMAD6 [15]. DHCR24 prevents apoptosis by reducing caspase-3 levels, particularly after oxidative stress [16]. GS755 and GS838 shared NT5DC1 (upregulated in GS755, downregulated in GS838) and LPAR2 (downregulated in GS755, upregulated in GS838), highlighting again the inter-tumoral diversity in transcriptomic changes upon treatment with TMZ + BMP4 (Figure 6b). These differential expressions of FIG4, RPS6KA4, DHCR24, and CDH10 were confirmed using qPCR (Supplementary Figure S3).

Interestingly, BHLHE40 was significantly upregulated in both GS627 and GS838; the encoded transcription factor stimulates proneural-to-mesenchymal transition in GBM [17]. Further pathway enrichment analysis revealed that other pathways were enriched in at least two cultures (Figure 6c). They include purine metabolism, N-linked glycosylation, positive regulation of the apoptotic signaling pathway, regulation of MAP kinase activity, and viral life cycle.

Thus, batch RNA-seq highlighted the inter-tumoral heterogeneity in response to treatment with TMZ only and certainly for BMP4 only, but also reveals potential mechanisms of action for the synergy of TMZ + BMP4. We propose that more elaborate studies of this type can aid and potentiate future clinical studies of this combinatorial therapy.

## 3. Discussion

In this study, we sought to define a more optimal paradigm for co-treatment of patients with GBM with TMZ + BMP4, as information from such studies could be pivotal for the design of future clinical trials. Furthermore, we aimed to estimate the proportion of patients, based on patient-derived GBM cell cultures, that might benefit from this combination therapy. Finally, we intended to provide a starting base towards better understanding of the underlying mechanisms, including the transcriptional level, of synergy of these two agents. The proposed mechanism underlying the sensitization of cultured GBM cells by BMP4 to TMZ, and, therefore, providing the rationale for BMP4 + TMZ combination, is that GSCs are differentiated by BMP towards an astroglial cell fate rather than achieving increased cell proliferation and that such cells would be more susceptible to chemotherapy. In this study, we focused on *IDH* wild-type glioma, since BMP4 is generally more downregulated than in *IDH* mutant glioma, [18] and, therefore, the potential effect size would be maximal. Moreover, including two types of tumors with different pathophysiology would have very likely confounded our results.

We first hypothesized that sequential treatment would be most effective. However, our results show that the simultaneous treatment was generally the most efficient in achieving the desired decrease in cell viability. We then applied this treatment strategy to a panel of 20 patient-derived cultures; about 70% of these were found to likely benefit more from this combination therapy than monotherapy (either BMP4 or TMZ). The cultures that did not benefit from combination therapy were generally those that were very sensitive to either of the monotherapies and, therefore, had little potential benefit from combinatorial treatment. The observed inter-tumoral heterogeneity (with regard to reduction in cell viability in the case of TMZ + BMP4) can also clarify the controversies in the literature regarding the synergy of TMZ and BMP4. Often, a very limited number (1–3 cultures) of tumors are used to assess therapy efficacy, which is not representative of the entire patient population and limits the generalization of such results [9,19].

We show that the decrease in cell viability when TMZ + BMP4 act synergistically is a result of increased cell death by annexin-V-detectable apoptosis. Contrary to cell cycle arrest, induction of apoptosis would have a more permanent effect in the treatment of GBM, provided feedback mechanisms—if these occur—do not install stimulated, increased GSC self-renewal in vivo.

RNA-seq analysis reveals that in cultures in which TMZ + BMP4 act synergistically, MAPK signaling is ultimately deregulated only upon combination therapy. BMPs have been shown to both stimulate and inhibit the MAPK signaling pathway, regardless of whether the involved kinases are components of non-SMAD signaling or co-act with SMAD signaling in BMP-exposed cells [20,21]. MAPK signaling is associated with resistance to TMZ, and inhibition of the p38-MAPK pathway can sensitize GBM cells to TMZ by suppressing BCL-2 and activating p-AKT [22].

Another enriched pathway upon combination treatment was the purine metabolism pathway. Shireman and co-workers reported that purine biosynthesis induces chemoresistance in GBM cells [23]. Further research is necessary to clarify how BMP4 inhibits the purine metabolism. N-linked glycosylation was also enriched in all three RNA-sequenced cultures. Protein glycosylation affects the majority of proteins and, thereby, many biological processes [24]. In high-grade gliomas, N-glycosylation is associated with malignant behavior and is regulated by histone acetylation-dependent mechanisms [25]. BMP4 is one of the factors that affect histone acetylation [26] and might potentially diminish the aggressive behavior of GBM when treated with TMZ. Further studies are necessary to elucidate the interaction between N-glycosylation and TMZ + BMP4 in GBM.

Some common DEGs encompass RPS6KA4, which is associated with decreased inhibition of BMP–SMAD signaling; DHCR22, leading to enhanced apoptosis; and BHLHE40, which enhances TGFß/mTOR-induced mesenchymal differentiation [27]. Aberrant mesenchymal differentiation of GSCs is associated with decreased chemosensitivity [28]. Therefore, further assessment into enhanced chemosensitivity in cells with upregulated BHLHE40 is necessary. In addition, each culture had a unique transcriptomic signature upon combination treatment with TMZ + BMP4, implying that multiple mechanisms in a variable genetic context are likely responsible for the increased apoptosis elicited by this combination therapy.

The transcriptomic signatures and the resulting DEGs should still be interpreted with caution, as we sequenced cultures that showed decreased viability after seven days of treatment. We performed sequencing after two days of treatment, since we did not observe a significant increase in annexin-V+ signals in any of these cultures at that time point, thus expecting that major apoptotic signatures would occur after longer treatment only. The lack of common DEGs in GBM tumors thus far can also still result from the limited number of RNA-sequenced cultures. Therefore, it would probably be ideal to sequence at least to 3–5 cultures with an equal response with regard to cell viability, proliferation, and apoptosis to BMP4, TMZ, and TMZ + BMP4. However, to find such an equally responsive tumor set, the initial discovery set should be greatly enlarged, all this owing to the higher inter-tumoral heterogeneity.

In addition, these signatures also need validation in samples of patients treated with TMZ + BMP4 if this combination treatment is to be tested in a clinical trial. We also have to acknowledge that these patient-derived cultures, even though cultured in serum-free conditions and assessed still at low passage number, cannot totally resemble the tumor microenvironment in each patient either.

Local delivery of BMP4 as tested in a small number of patients with recurrent GBM, was recently shown to be safe [10]. This present study suggests that co-treatment with TMZ would increase the proportion of patients who could benefit from either one of these treatments as a combination therapy and when effective would significantly enhance the desired apoptotic potential of these drugs. We acknowledge that combination therapy of radiotherapy, temozolomide, and BMP4 within the current golden standard treatment paradigm would be logistically challenging. However, combination therapy in contrast to sequential therapy would also be the most time-efficient therapy and can be well combined with the adjuvant rounds of TMZ that are also part of standard treatment protocol. As with all therapies that have been described for GBM patients, improved tumor stratification is, in any case, necessary to reach the appropriate responsive patient subcategory especially since (albeit in a small subset of tested patients) TMZ + BMP4 sometimes has an antagonistic effect. Since sensitivity to monotherapy is not predictive of sensitivity to combination therapy, novel predictive markers (and their steady-state transcript levels) are still needed for proper patient selection. Here, we describe for the first time the synergistic effects of BMP4 with TMZ in a set of 20 patient-derived samples and with an optimized treatment paradigm. We believe it can help future studies in discovering and improving predictive therapeutic markers and demonstrating their clinical value in future combination therapies.

## 4. Materials and Methods

### 4.1. Cell Culture

Resected tumor samples from 20 patients were collected by the Department of Neurosurgery of the Erasmus Medical Center (Rotterdam, NL) after obtaining informed consent in accordance with protocols approved by the institutional review board. After mechanical dissociation the tissues were enzymatically dissociated using DNase and collagenase. The cells were cultured in serum-free medium as previously described [29].

### 4.2. Cell Viability Assay

Cell viability was assessed after seven days of treatment with 60 ng BMP4/mL (B&D systems, Minneapolis, MN, USA; see also [4]), 120 µM TMZ (an optimal dose to discriminate between sensitive and resistant cultures [12], Sigma, Kanagawa, Japan), or a combination of both. Lyophilized BMP4 was first dissolved in 4 mM HCl-0.1% BSA. The CellTiter-Glo kit (Promega, Madison, WI, USA) was used to quantify cell viability. Luminescence was measured using a Tecan Infinite200 Pro-reader (Tecan Life Sciences, Männedorf, Switserland). The average cell viability with standard deviation was normalized to that of the untreated cells.

### 4.3. Synergy Analysis

Statistical determination of synergy was performed in R version 4.0.5 [30] using the Synergyfinder package [31]. The used reference model is the highest single agent (HSA) model [32], which assumes that the expected combination effect is the maximum of the monotherapy response at the respective concentration. We considered a value of >10 as synergistic, between −10 and +10 as additive, and <−10 antagonistic.

### 4.4. EdU Incorporation Assay

Cells seeded in glass-bottomed plates were treated for two days with 60 ng BMP4/mL, 120 µM TMZ, or a combination of both. Subsequently, they were incubated with EdU labeling reagent (EdU Staining Proliferation Kit iFluor 647, Abcam, Cambridge, UK) for three hours and processed according to the manufacturer’s protocol. Counterstaining of nuclei was performed using Hoechst 33342. Three images per biological replicate were captured on the Leica TCS SP5 microscope (Leica Microsystems, Amsterdam, The Netherlands). The percentage of EdU-positive (+) nuclei was determined using the ImageJ software version 1.54 [33]. Average and standard deviation of the EdU+ cells were calculated.

### 4.5. Annexin-V Assay

Cells treated for four days with BMP4, TMZ, and BMP4 + TMZ (at the aforementioned concentrations) were detached using Accutase (Invitrogen, Carlsbad, CA, USA). Cells were stained with Annexin V-iFluor 555 (Apoptosis Staining/Detection Kit, Abcam) and Hoechst 33342 according to the manufacturer’s protocol. Fluorescence was measured on a BD LSRFortessa flow cytometer and analyzed using FlowJo TM v10.6.2. The average fraction and standard deviation of annexin V-positive (+) cells per biological replicate were calculated.

### 4.6. cDNA Library Preparation and Sequencing

A total of 24 h after seeding, cells were treated with 60 ng BMP4/mL, 120 µM TMZ, or BMP4 + TMZ for 48 h. The cells and untreated controls were detached using accutase and washed with PBS. RNA was extracted using TRIzol (Thermo Fisher Scientific, Waltham, MA, USA) as previously described [34]. RNA was quantified using a NanoDrop 1000 (Thermo-Fisher Scientific) and diluted to 500 ng/mL, and the quality was assessed on the Agilent 2100 Bioanalyzer; RNA integrity number of each used sample was at least 8.

Three biological replicates of each sample were used for library preparation using (Illumina TruSeq Stranded mRNA Library Prep Kit, Illumina, San Diego, CA, USA). The resulting cDNA libraries were sequenced according to the Illumina TruSeq Rapid v2 protocol using an Illumina HiSeq2500 sequencer (Illumina, San Diego, CA, USA). Reads of 50 base pairs were generated and mapped against the GRCh38 reference genome using HiSat2 (v2.1.0) and gene expression values were called using htseq-count (v0.11.2).

### 4.7. RNA-Seq Bioinformatics Analysis

Differential gene expression (DEG) analysis was performed using the edgeR package [35] and values were adjusted for multiple testing using the Benjamini and Hochberg method [11]. Genes with a *p*-value < 0.01 and a log-fold-change of >2 were considered as significant DEGs. Gene set enrichment analysis (GSEA) of the DEGs was performed using Metascape version 3.5 [13].

### 4.8. Quantitative PCR

Total RNA was isolated using TRIzol reagent [34]. cDNA was synthesized using RevertAid (Thermo Fisher Scientific). Real-time qPCR was performed using the Bio-Rad CFX96 system; for primers, see Table 1. All qPCR experiments were conducted in triplicate.

### 4.9. Statistics

Descriptive statistics were calculated using GraphPad Prism version 8.2.1. Means were compared using Student’s *t*-test or Dunnett’s test for multiple comparisons. α < 0.05 was considered statistically significant.

## Figures and Tables

**Table 1:** List of primers used.

Primer	Sequence
GAPDH for	5′-AATCCCATCACCATCTTCCA-3′
GAPDH rev	5′-CATCATGCAGCACCTCAGGT-3′
RPS6KA4 for	5′-GATCACAGAAGCCAACCTG-3′
RPS6KA4 rev	5′-GAAGTTCTCCACGCTCAC-3′
DHCR24 for	5′-CCGTGGTTCTTTAAGCATGT-3′
DHCR24 rev	5′-AAAGGGGATAATGTCCTGGAG-3′
FIG4 for	5′-TCTGTATGAGACTAGAGCTAGATA-3′
FIG4 rev	5′-CTGCATTATTGCTCCCAACT-3′
CDH10 for	5′-CATTCGAGTGTGTGCTTGT-3′
CDH10 rev	5′-TGCAAACAGTACTACTATAACCAG-3′
BHLHE40 for	5′-GGAGACCTACCAGGGATGTA-3′
BHLHE40 rev	5′-TATTCCCCGTCTTGACTTGT-3′
LPAR2 for	5′-ACACCCGCATTTTCTTCTAC-3′
LPAR2 rev	5′-GAACGCCCCCAGGATG-3′
NT5DC1 for	5′-GCCATCTCTGGATAAACCTG-3′
NT5DC1 rev	5′-ACCAAAATAAACAACCTTGGGT-3′

## Data Availability

The RNA-sequencing data generated in this study are publicly available in the European Genome–Phenome Archive (EGA) at EGAS00001007095.

## Supplementary Materials

The supporting information can be downloaded at: https://www.mdpi.com/article/10.3390/ijms251810176/s1.
