# Supplementary material for: BMP4 and Temozolomide Synergize in the Majority of Patient-Derived Glioblastoma Cultures
Source: Int J Mol Sci. 2024 Sep 22;25(18):10176. doi: 10.3390/ijms251810176 (PMC11432198; doi:10.3390/ijms251810176)
Supplement: Supplementary file 1 [file ijms-25-10176-s001.zip › Supplementary figures.pdf]

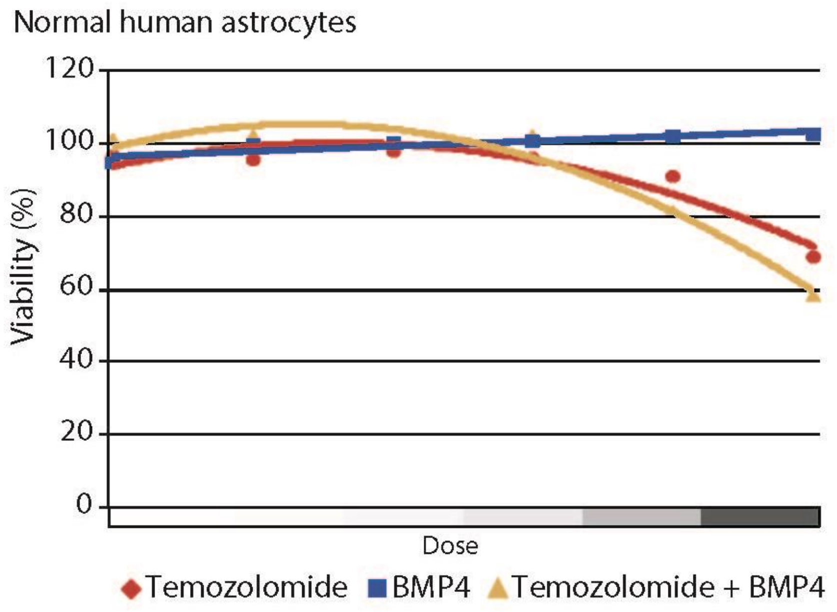

<u>Supplementary figure S1</u>
Viability of normal human astrocytes after 7 days of treatment with a 3-fold dilution ranging from 180 ng down to 0.74 ng BMP4/ml (blue) and 360 µM down to 1.5 µM TMZ (red) and a combination of both (yellow).

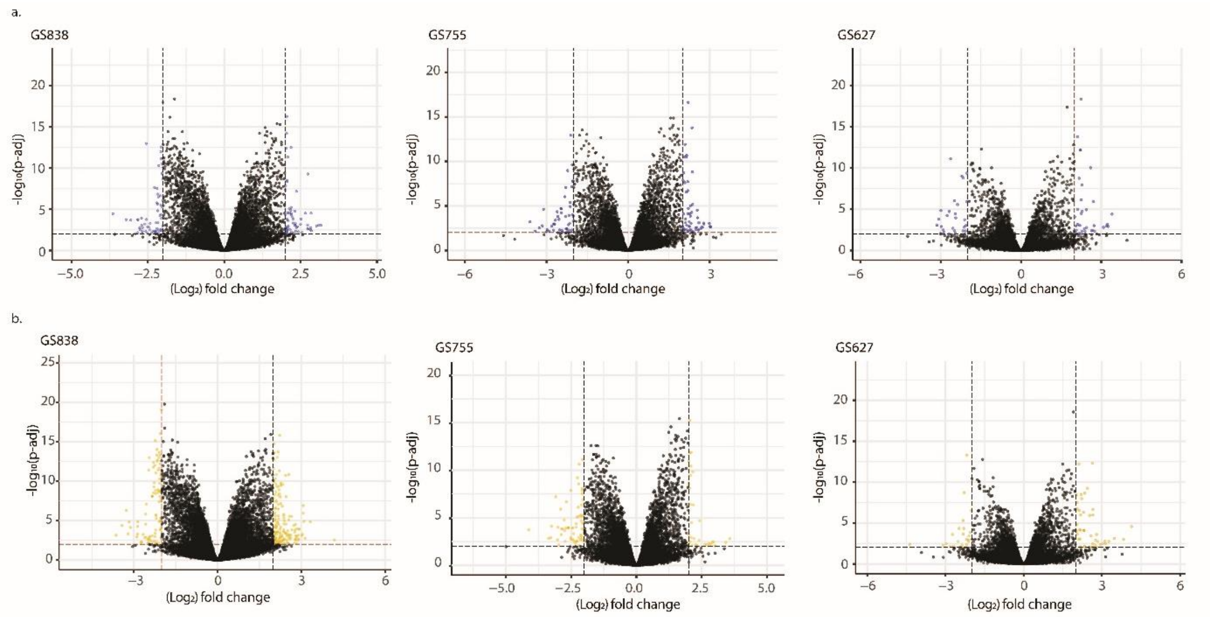

<u>Supplementary figure S2</u>
Volcano plot of gene expression after (a) BMP4 (genes unique to BMP4 in blue) and after (b) TMZ+BMP4 (genes unique to combination treatment in yellow).

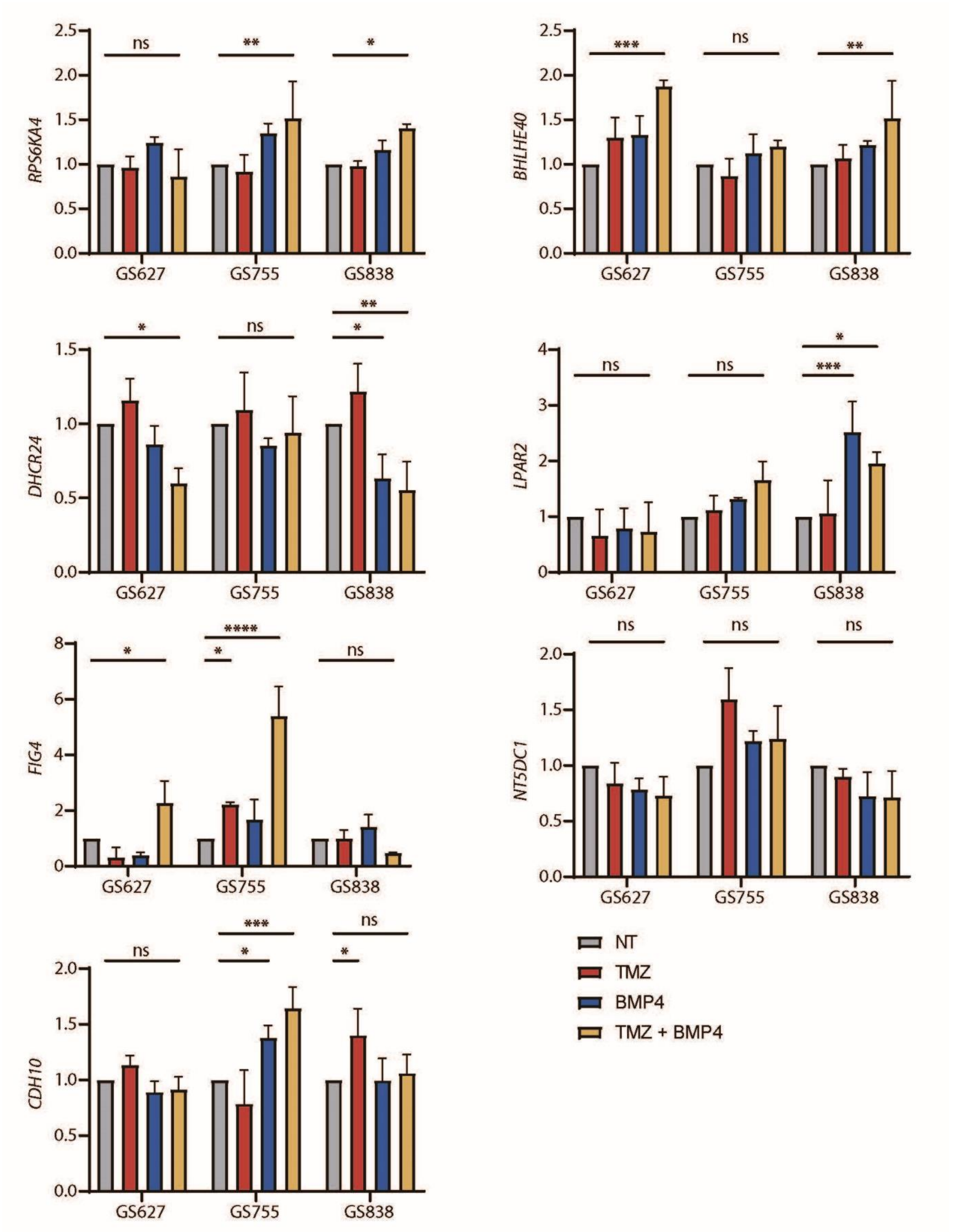

Supplementary figure S3

Bar plots of by qPCR validated differentially expressed genes unique to TMZ + BMP4 with expression values normalized to *GAPDH.* Grey = non-treated (NT), red = temozolomide (TMZ), blue = Bone Morphogenetic protein 4 (BMP4), yellow = TMZ + BMP4. *p<0.05, **p <0.01, ***p<0.001, ns = not-significant.
