# Supplementary material for: BMP4 and Temozolomide Synergize in the Majority of Patient-Derived Glioblastoma Cultures
Source: Int J Mol Sci. 2024 Sep 22;25(18):10176. doi: 10.3390/ijms251810176 (PMC11432198; doi:10.3390/ijms251810176)
Supplement: Supplementary file 1 [file ijms-25-10176-s001.zip › Supplementary table S1.pdf]

Supplementary table S1

Genetic background of used primary cultures

Culture	Gender	Recurrent	MGMT	PTEN	IDH	EGFR/chr 7	1p19q	TP53
GS755	M	N	Unmethylated	Mutation	WT	Amplification	WT	Mutation
GS838	M	Y	Unmethylated	Mutation	WT	Amplification	WT	WT
GS786	M	N	Methylated	Loss	WT	Amplification	1p imbalance	WT
GS607	M	N	Unmethylated	Loss	WT	Amplification	19q imbalance	Mutation
GS799	M	N	Methylated	Loss	WT	Amplification	1p19q loss	Mutation
GS502	F	N	Methylated	Loss	WT	Amplification	19q imbalance	Mutation
GS916	M	Y	Unmethylated	Loss	WT	Amplification	1p loss	WT
GS824	M	N	Methylated	Loss	WT	Amplification	WT	WT
GS627	F	N	Unmethylated	WT	WT	Amplification	19q imbalance	WT
GS832	M	N	Unmethylated	Loss	WT	Amplification	19q imbalance	WT
GS851	M	N	Methylated	WT	WT	Amplification	19q imbalance	WT
GS741	M	N	Unmethylated	Loss	WT	Amplification	WT	Mutation
GS636	M	N	Unmethylated	Loss	WT	Amplification	WT	WT
GS622	F	N	Methylated	Loss	WT	Amplification	1p loss	Mutation
GS630	M	N	Unmethylated	Imbalance	WT	WT	WT	WT
GS784	F	Y	Unmethylated	Mutation	WT	Amplification	1p imbalance	WT
GS820	F	N	Methylated	Loss	WT	Amplification	1p19q imbalance	WT
GS968	M	N	Methylated	Loss	WT	Amplification	1p loss	WT
GS802	M	N	Unmethylated	Loss	WT	Amplification	WT	Mutation
GS612	M	N	Unmethylated	Mutation	WT	Amplification	WT	WT
